# Does Isoniazid Preventive Therapy Provide Better Treatment Outcomes in HIV-Infected Individuals in Northern Ethiopia? A Retrospective Cohort Study

**DOI:** 10.1155/2020/7025738

**Published:** 2020-01-21

**Authors:** Tesfay Mehari Atey, Helen Bitew, Solomon Weldegebreal Asgedom, Asrat Endrias, Derbew Fikadu Berhe

**Affiliations:** ^1^Clinical Pharmacy Unit, School of Pharmacy, College of Health Sciences, Mekelle University, Mek'ele, Ethiopia; ^2^Department of Pharmacognosy, School of Pharmacy, College of Health Sciences, Mekelle University, Mek'ele, Ethiopia; ^3^Medical Biochemistry, Biomedical Institute, College of Health Sciences, Mekelle University, Mek'ele, Ethiopia; ^4^Department of Pharmacology, School of Pharmacy, College of Health Sciences, Mekelle University, Mek'ele, Ethiopia

## Abstract

**Objectives:**

Early antiretroviral therapy (ART), isoniazid preventive therapy (IPT), and isoniazid-rifapentine (3HP) are effective strategies for preventing tuberculosis (TB) among people living with HIV (PLHIV). The study aimed to determine the effect of IPT on the TB incidence, follow-up CD4^+^ T cells, and all-cause mortality rate. *Participants*. Eligible patients on ART (*n* = 1, 863) were categorized into one-to-two ratios of exposed groups to IPT (*n* = 621) and nonexposed groups to IPT (*n* = 1, 242). Exposed groups entered the cohort at their first prescription of IPT, and unexposed groups entered into the study at the first prescription of ART and then followed until the occurrence of the outcome or date of administrative censoring (June 30, 2017). The outcome endpoints were TB incidence, follow-up CD4^+^ T cells, and all-cause mortality rate.

**Results:**

The follow-up CD4^+^ T cells for the exposed and nonexposed groups were 405.74 and 366.95 cells/mm (World Health Organization (WHO), 2017), respectively, a statistically significant finding (*t*_1861_ = −3.770, *p* < 0.0001; Cohen's *d* = 0.186). Nine percent of the exposed patients (620 incidence of TB per 100,000 person-years (PYs)) and 21.9% of the nonexposed patients (3160 incidence of TB per 100,000 PYs) developed TB. Mortality rate (per 100,000 PYs) was 440 for the exposed and 1490 for the unexposed patients. Statistically significant determinants of the all-cause mortality were unscheduled follow-up (AHR = 1.601; 95% CI: 1.154–2.222) and unable to work properly (AHR = 2.324; 95% CI: 1.643–3.288).

**Conclusion:**

This study demonstrates the effect of IPT in reducing incidence of TB and all-cause mortality rate and improving follow-up CD4^+^ T cells. Promoting IPT use can help to achieve the TB eradicating national agenda in Ethiopia.

## 1. Introduction

As one of the most serious opportunistic infections (OIs) among people living with HIV/AIDS (PLHIV), tuberculosis (TB) often leads to mortality in low-income African countries [1, 2]. HIV/AIDS accelerates the reactivation of latent TB infection (LTBI) and rapid progression to primarily TB [3–7], and vice versa [7]. The progression of LTBI to active disease during the lifetime of the general population is about 5–10%, compared to 30% among PLHIV coinfected with TB [6, 8, 9]. This rapid progression is more likely to happen within the first years of infection [10].

The 2016 global report estimated about 6.3 million incident cases of TB, equivalent to 61% of the estimated incidence of 10.4 million. There were 476,774 reported cases of HIV/TB coinfections, in which 85% were on antiretroviral therapy (ART) [3]. The prevalence of LTBI included about a quarter to one-third of the global population [7, 11]. Rates of LTBI are 49% in Uganda [12], 55.2% in South Africa [13], and ranged from 31.2% to 64% in Ethiopia [14–16]. In Tigray (Northern Ethiopia), the prevalence of HIV was 4.7% among adults [17] and the number of PLHIV in the region is estimated to be 117,976 even though the effort at reduction of mortality is not adequate. The overall weighted prevalence of bacteriologically confirmed pulmonary TB in the Tigray region was found to be 216/100,000 [18].

Some preventive strategies such as ART, IPT, and isoniazid-rifapentine (3HP) are required to reduce the risk of LTBI progression to active disease to achieve the 2035 target of eliminating TB [10, 19–21]. Several studies have explored the effect of IPT on mortality [22–25]. However, coverage and uptake of IPT is not 100% [2], and IPT coverage in the region was estimated to be 20% in which fear of creating isoniazid resistance and lack of commitment of health managers to scale-up the program were some of the main barriers hindering implementation of IPT [26].

The effect of IPT together with ART on the CD4^+^ T cells and rate of mortality among PLHIV on chronic HIV care follow-up is poorly understood with conflicting results. Reduction of all-cause mortality was not evidenced in some studies [8, 19]. The use of IPT among PLHIV is associated with a significant reduction in incidence of TB [19]. Although the TB-related mortality benefits were revealed by randomized controlled trials of IPT, all-cause mortality reduction was not significantly shown [27, 28]. In addition, the applicability of the data obtained from other context could have limitations due to the variability in clinical cohorts in different populations. IPT is also largely affected by the population genetics. Hence, the primary aim of the present study was to determine whether IPT lowers the incidence of TB and rate of all-cause mortality and improves the CD4^+^ T cells independent effect of ART on the incidence of TB, CD4^+^ T cells, and mortality among PLHIV in Tigray region, Ethiopia.

## 2. Methods

### 2.1. Study Settings

A regional-based propensity-matched retrospective cohort study was conducted in six ART clinics of selected hospitals in Northern Ethiopia, Tigray Regional State. We selected six sites, namely, Alamata Hospital, Ayder Comprehensive Specialized Hospital, Lemlem Karl Hospital, St. Marry Hospital, Suhul Hospital, and Wukro Hospital, randomly from the 14 hospitals [29] using a lottery method. World Health Organization guidelines have expanded the recommended criteria for life-saving ART eligibility among people with HIV infection, though major changes in ART eligibility criteria have been observed during different periods including 2010, 2013, and 2016 [30].

### 2.2. Sample Size and Sampling Technique

The Kelsey method was employed in the sample size determination [31]. Accordingly, we used 5% for two-tailed type-I error, 80% power, two-sided 95% confidence interval (CI), unexposed to exposed ratio = 2 : 1, and 0.75 for minimum risk ratio to detect. From a previous study, 10% prevalence and 4.5% prevalence of mortality among unexposed and exposed groups, respectively, were reported [25]. Adding a design effect of 1.5, a total of 621 patients were considered as exposed groups. Doubling this number, 1,242 subjects were included in the nonexposed group with a total of 1,863 patients.

**Figure 1:**
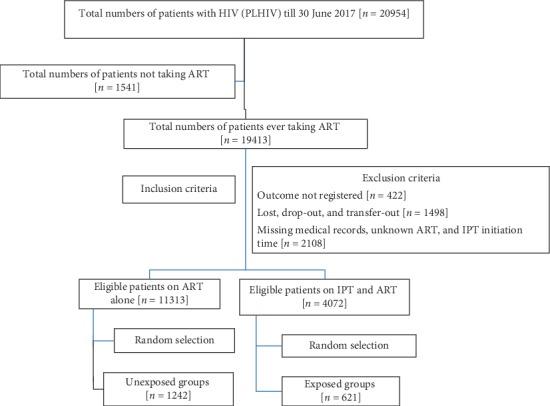
A flow diagram showing selection of study patients on IPT plus ART and ART alone.

**Figure 2:**
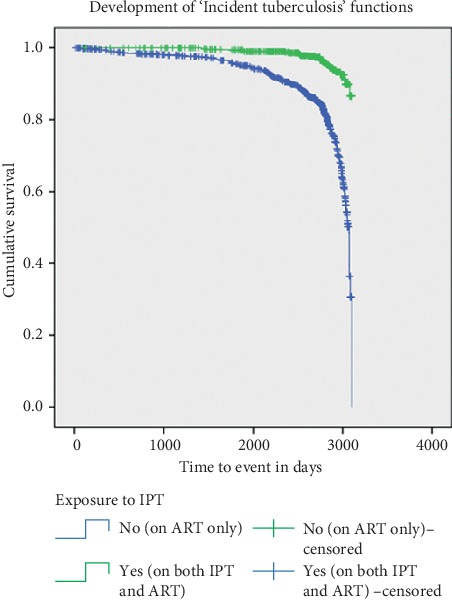
Kaplan–Meier for incidence of TB among patients living with HIV/AIDS taking IPT and ART versus ART alone in Northern Ethiopia, 2009–2017.

A two-stage sampling technique, stratified sampling technique followed by a simple random sampling technique, was employed. From the total of 1,863 study participants, 310 patients were stratified into the six ART clinics. These 310 study patients were selected using a computer-generated simple random sampling technique at each clinic who started their ART and/or IPT starting from the index period using their unique ART identification number. Out of these 310 study patients per a clinic, 103 exposed patients were first randomly selected from patients taking IPT and ART. For every exposed patient, two unexposed patients (on ART alone) were randomly selected after adjustment for the WHO clinical stage, baseline CD4^+^ T cells count, treatment categories, and adherence status (Figures 1 and 2).

### 2.3. Inclusion and Exclusion Criteria

Eligible patients were selected based on the following criteria: PLHIV having at least two or more visits starting from the index period and receiving their healthcare at the clinics. On the contrary, PLHIV were on anti-TB treatment before the enrollment, with altered or elevated liver function tests at baseline (five times higher than the upper normal limits in asymptomatic patients or three times above the upper normal limits in symptomatic patients), with adherence rate of <85% or missed ≥6 doses out of 30 doses, and with unknown period (month and year) of starting both regimens and treatment outcome were excluded from the study (Figure 1 and Supplementary Figure 1). Determination of TB incidence, biannual CD4^+^ T cells counts, and mortality status was the treatment outcome variables.

### 2.4. Data Collection

A customized data collection form was adopted from the format of Federal Ministry of Health. The data abstraction form was piloted before implementation, and 12 data collectors, who were recruited from the staff members of the hospital and trained as data collectors for two days, collected the data. The patients' medical records were followed until occurrence of the outcome (incidence of TB and mortality) or date of administrative censoring (end period of data collection, i.e., June 30, 2017). Completeness of the collected data was supervised and monitored adequately by the investigators and supervisors during the data collection process. The duration of IPT is 6 months, and it is given at a daily dose of 5 mg/kg (maximum 300 mg). IPT and ART visits were given as integrative services at the clinic.

### 2.5. Operational Definitions

Unscheduled follow-up was defined when the participant did not attend at least one routine follow-up visit on the appointment date. Otherwise, the participant was deemed to have a scheduled follow-up.

The index period was identified when exposed groups entered the cohort at their first prescription of IPT, and unexposed groups entered into the study at the first prescription of ART.

Diagnosis of TB was done according to the Ethiopian Tuberculosis, Leprosy and TB/HIV Prevention and Control Program Manual [32], where all suspects of any form of TB must be examined according to the standardized diagnostic procedures in which the microscopic examination of sputum is the most important and reliable one, followed by radiological investigation, acid fast bacilli (AFB) culture, and histopathology. The identification of TB suspects (cough for more than two weeks) and followed by screening them by examination of sputum smears are performed during every visit or at every suspicion of TB.

### 2.6. Data Analysis

Data were analyzed using Statistical Package for Social Sciences (SPSS® Statistics) program version 21 (SPSS; Chicago, IL, USA), STATA® version 12, and R®-software (R i386 2.15.3). Kaplan–Meier (KM) analyses were used to compare the mortality rates and TB incidence-free survival time between the two cohorts. The assumption for proportional hazard was assessed graphically by log minus log survival curve (Supplementary Figure 2) and a time-dependent Cox-model (*p*-value for T_COV = 0.778). A sensitivity analysis, using propensity score matching, was performed to evaluate and address confounding by indication (Appendix in Supplementary materials).

An independent samples *t*-test was used to compare the follow-up CD4^+^ T cells between the two cohorts. Moreover, TB incidence and incidence rates of mortality per 100,000 persons-years (PYs) of follow-up were employed. We used exact dates to calculate person-years by summing of total time contributed by all subjects. In the propensity-matched cohort, an adjusted Cox proportional hazard model was repeated to find out the effects of sociodemographics, clinical characteristics, and treatment categories on the outcome variables. Cohen's *d* was used as an index for determining the strength of the effect size. In all the analyses, significance testing was done using two-sided *p* values and 95% CI, and a *p* value <0.05 was considered as statistically significant. Lastly, missing data for continuous variables were accounted by the expectation maximization model.

### 2.7. Patient and Public Involvement

This study was conducted in medical records of patients using standardized formats prepared by the Ethiopian Federal Ministry of Health, and hence, there was no direct involvement of the patients and the public.

### 2.8. Ethical Considerations

An ethical clearance was obtained from “Health Research and Ethics Review Committee” of College of Health Sciences (Reference number: ERC 0877/2016), Mekelle University. In addition, the Research Committee of Tigray Regional Health Bureau granted ethical permission to conduct the study (Reference number: 4366/7767/09). Permission to utilize patient data was also obtained officially from the administrative offices of the selected hospitals. To ensure confidentiality, name and other identifiers of patients, physicians, and other staff members of the centers were not recorded on the data abstraction instrument.

This study was funded by Mekelle University (Grant Number: CRPO/CHS/SM/015/09). The funder of the study had no role in study design, data collection, data analysis, data interpretation, or writing of the report. The corresponding author had full access to all the data in the study and had final responsibility for the decision to submit for publication. Lastly, this study was done in accordance with STROBE cohort reporting guidelines [33].

## 3. Results

### 3.1. Sociodemographic and Clinical Characteristics

Nearly one-third of females (34.9%) and males (30.9%) were exposed to IPT. Comparably, about one in three of the participants in the age group of 30–44 (34.1%) and participants dwelling in urban areas (33.5%) were exposed to IPT. Approximately one-third of the patients had a working functional status (33.7%), and those participants with a WHO clinical stage III at the baseline (38.9%) were exposed to IPT (Table 1).

In follow-up, 61.4% of the visits were scheduled and 38.6% of the visits were unscheduled. The average baseline body weight was almost similar in both groups: exposed patients (49.8 kg) and unexposed patients (49.2 kg). The average baseline BMI was also about 19.8 kg/m^2^ for both cohorts. Over the nine-year period, the weight was increased by about 6.6 kg for both cohorts; and the BMI was increased by 2.8 kg/m^2^ for the exposed group and by 3.8 kg/m^2^ for the unexposed group, interpreting into about 15% and 16% increase from baseline to 2017, respectively (Supplementary Figure 3).

Nearly one-third (32.31%) of the patients were initiated with d4T + 3TC + NVP followed by AZT + 3TC + NVP, in which this regimen was taken by about a quarter of the patients (24.3%). Majority of the study participants (93.5%) were on the first line ART regimen. The mean duration on ART for both groups was 6.8 years. Furthermore, the average time between ART initiation and IPT initiation is 239 days.

### 3.2. Effect of IPT on Tuberculosis

From the total patients, 16.1% of them developed TB, in which 9.3% of the exposed groups and 21.9% of the unexposed groups developed TB (*p* < 0.0001) (Table 2).

### 3.3. Effect of IPT on CD4^+^ T Cells

**Figure 3:**
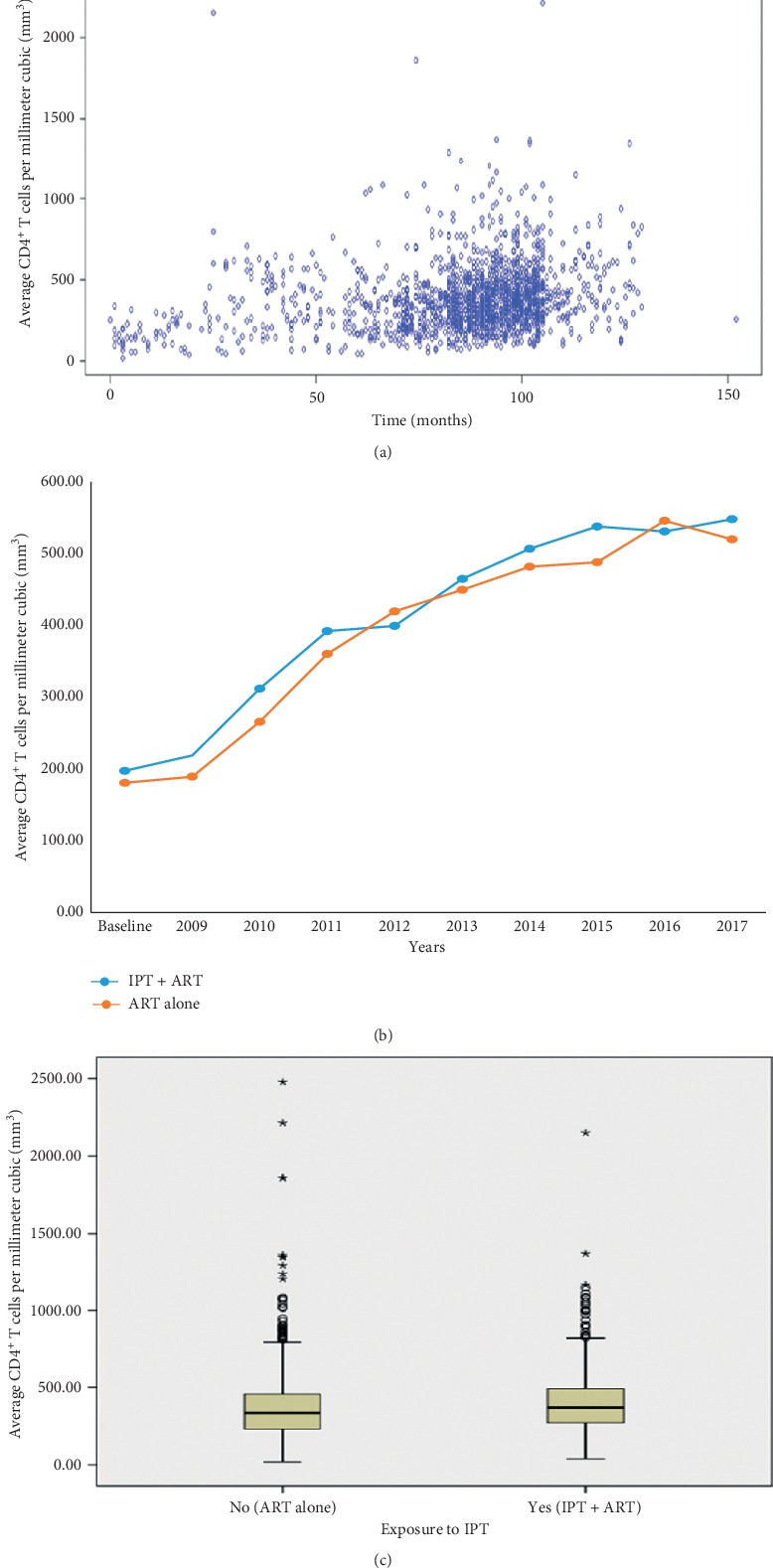
Trend of CD4^+^ T cells per millimeter cubic among patients living with HIV/AIDS taking IPT and ART versus ART alone in Northern Ethiopia, 2009–2017. (a) Overall CD4^+^ T cell trends; (b) trend comparison by cohorts; (c) mean CD4^+^ T cells comparison by cohorts. AIDS: acquired immune deficiency syndrome; ART: antiretroviral therapy; CD4+ T cells: cluster of differentiation 4 positive T cells; HIV: human immunodeficiency virus; IPT: isoniazid preventive therapy.

The mean follow-up CD4^+^ T cells (in cells per mm^3^) were 405.74 for IPT exposed patients and 366.95 for IPT unexposed patients. For the exposed group, the CD4^+^ T cells showed increase by 177.61% (i.e., 344.81 cells per mm^3^) over the past nine years, with an average of 538.94 in 2017, up from a mean of 194.14 at the baseline. Likewise, for the second cohort, the CD4^+^ T cells showed increase by 187.67% (i.e., 333.58 cells per mm^3^) over the past nine years, with an average of 511.33 in 2017, up from a mean of 177.75 at the baseline (Figure 3).

After propensity score matching, there was a statistically significant difference in mean CD4^+^ T cells between the two cohorts (*t*_1861_ = −3.770, *p* < 0.0001). The average CD4^+^ T cells for patients not exposed to IPT (mean = 366.951 cells/mm [3]; SD = 209.827 cells/mm^3^) was 38.792 cells/mm^3^ (95% CI: −58.970 to −18.614 cells/mm^3^) lower than the average CD4^+^ T cells for patients exposed to IPT (mean = 405.742 cells/mm [3]; SD = 208.348 cells/mm [3]; Cohen's *d* = 0.186). The mean CD4 cells in different years are given in Table 3.

**Figure 4:**
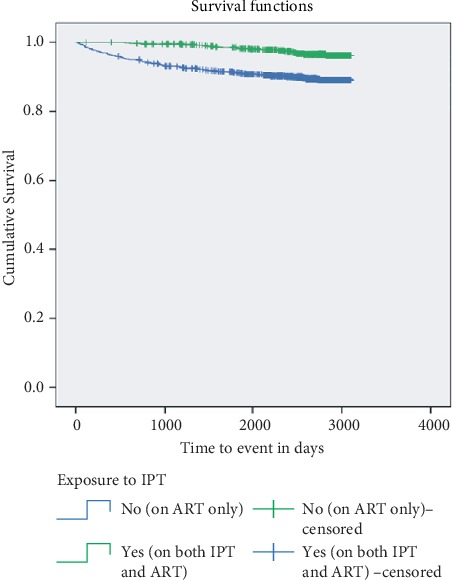
Kaplan–Meier survival analysis for patients living with HIV/AIDS taking IPT and ART versus ART alone in Northern Ethiopia, 2009–2017.

The total person-years (PYs) of observation for patients exposed to IPT and for those not exposed to IPT were 4,512 and 8,567, respectively. The incidence rate of mortality was 440 per 100,000 PYs (incidence rate difference = −1050; 95% CI: −1270 to −730 per 100,000 PYs; *p* value < 0.0001) for the exposed groups and 1490 per 100,000 PYs (incidence rate ratio = 3560; 95% CI: 2100 to 3980 per 100,000 PYs) for the unexposed groups. The KM analysis for survival function illustrated that exposed patients had a better survival chance than unexposed patients at all levels of time (*p* < 0.0001) (Figure 4).

Statistically significant preventive factors from mortality were urban residence compared to rural residence (AHR = 0.595; 95% CI: 0.427–0.831), CD4+ T (in cells per mm^3^) ≥ 250 compared to <250 (AHR = 0.217; 95% CI: 0.154–0.307), and exposure to IPT (AHR = 0.339; 95% CI: 0.211–0.545). On the other hand, unscheduled follow-up (AHR = 1.601; 95% CI: 1.154–2.222) and unable to work properly (AHR = 2.324; 95% CI: 1.643–3.288) were statistically associated with mortality (Table 4).

## 4. Discussion

The use of IPT with ART regimen significantly reduced all-cause mortality and incidence of TB and improved CD4^+^ T cells, as evidenced in this study. The mortality benefit due to the initiation of IPT is evidenced in other studies conducted in Ethiopia [25], Tanzania [34], and South Africa [35]. Moreover, the initiation of IPT was significantly associated with reduced TB-caused mortality [36–38], all-cause mortality [39, 40], and illness among PLHIV [41].

Without this protective effect, an increased hazard of TB on mortality was described [42] as HIV-related TB is the leading infectious cause of death worldwide [43]. However, the use of IPT in reduction of mortality was not always found to be significant, as reported in the previous studies [8, 27, 36, 44–46].

The incidence of TB is found to be about five times more among unexposed groups compared to the exposed cohort, which is in line with a chemoprophylaxis study done in Brazil that provided similar evidence [8]. One cohort study described 11 cases of TB (among 316 patients observed during a two-year follow-up) with the incidence of TB in the IPT group reported to be 3.5 cases per 100 persons per year [47]. In another study, eight of 130 HIV-infected patients who received IPT for nine months developed TB during a 43-month follow-up period [48].

Effectiveness of IPT on new incidence of TB has been suggested in different local studies from Ethiopia and in other countries such as South Africa, Uganda, and Ivory coast [25, 36, 48–52]. Indeed, some studies from Ethiopia and South Africa involved PLHIV who have not started ART [25, 35]. Despite IPT's impact against new development of TB, IPT's long-term protection against TB is still ambiguous. Many African studies stated that the chemoprophylaxis prevented PLHIV from TB while receiving IPT only. Immediately after IPT's duration of action, its protection effect was found to be weakened [40, 44, 53–57]. On the contrary, studies conducted in US, Mexico, and Brazil did not find any waning effect of TB protection [44, 49, 51]. On the contrary, Rangaka et al. study done in South Africa found a steady rise of TB incidence rate after one year of IPT termination [57].

About twice as many patients residing in rural areas were experiencing death compared to patients living in urban areas. This finding may be tied to the presence of less follow-up, care and treatment; lesser awareness regarding the complications of the disease and OIs; and higher preponderance to lost and drop-out for patients residing in rural areas compared to urban areas. On the contrary, patients whose follow-up was scheduled were less likely to die than patients whose follow-up was unscheduled, and on-time scheduling may be associated with good adherence to the long-term medications.

There is fivefold more risk of death among patients with low CD4 count compared to patients with higher CD4 count. Several other studies are consistent with this finding, and they also pointed to the fact that severe immune suppression is observed in those with CD4+ T cell count below 200 cells/mm^3^ [58, 59]. Institutional-based cross-sectional study conducted in Ethiopia reported that 79.5% of TB infection occurred in low CD4^+^ T cells level (<200 cells/mm^3^). This increases morbidity and hastens progression of HIV to AIDS and mortality [60].

## 5. Conclusions

This study demonstrates the effect of IPT, when used with ART, in reducing incidence of TB and all-cause mortality rate and improving CD4^+^ T cells in Tigray region. Rural residence, lower CD4+ T cell values, nonexposure to IPT, and having unscheduled follow-up were independent predictors of increased likelihood of the all-cause mortality. Moving forward, we call for the healthcare providers to closely monitor the treatment outcome of patients taking ART alone and place a special effort in optimizing the outcome. Promoting IPT use can help achieving the TB eradicating national agenda in Ethiopia.

## Figures and Tables

**Table 1:** Baseline sociodemographic and clinical characteristics of patients living with HIV/AIDS in Northern Ethiopia, 2009–2017.

Variables		Exposure to IPT	
		No (ART alone)	Yes (IPT and ART)
Sex	Male	500 (69.2%)	223 (30.8%)
	Female	742 (65.1%)	398 (34.9%)
Age (in years)	<15	75 (63.0%)	44 (37.0%)
	15–29	235 (70.4%)	99 (29.6%)
	30–44	698 (65.9%)	361 (34.1%)
	45–59	210 (66.5%)	106 (33.5%)
	≥60	24 (68.6%)	11 (31.4%)
Residence	Rural	412 (67.0%)	203 (33.0%)
	Urban	930 (66.5%)	418 (33.5%)
Functional status	Working	827 (66.3%)	420 (33.7%)
	Ambulatory	318 (66.4%)	161 (33.6%)
	Bedridden	97 (70.8%)	40 (29.2%)
World Health Organization clinical staging	Stage I	178 (63.1%)	104 (36.9%)
	Stage II	251 (61.1%)	160 (38.9%)
	Stage III	649 (68.7%)	296 (31.3%)
	Stage IV	164 (72.9%)	61 (27.1%)

AIDS: acquired immune deficiency syndrome; ART: antiretroviral therapy; HIV: human immunodeficiency virus; IPT: isoniazid preventive therapy.

**Table 2:** Incidence of tuberculosis among patients living with HIV/AIDS taking IPT and ART versus ART alone in Northern Ethiopia, 2009–2017.

Disease/exposure		Exposure to IPT		Total	*p* value
		No (on ART only)	Yes (IPT and ART)		
Development of tuberculosis	No	970 (78.1%)	593 (95.5%)	1563 (83.9%)	<0.0001
	Yes	272 (21.9%)	28 (4.5%)	300 (16.1%)	
	Total	1242 (100.0%)	621 (100.0%)	1863 (100.0%)	<0.0001
Incidence rate of TB (per 100,000 persons-years)		3160	620		

On average, the time to development of TB incidence post-IP exposure was 926 days (two and half years). The incidence rate of TB (per 100,000 PYs) was 620 (incidence rate difference = −2250; 95% CI: −3000 to −2110; *p* value < 0.0001) for the exposed groups and 3160 (incidence rate ratio = 2340; 95% CI: 1530 to 3470) for the unexposed groups (Table 2). The KM analysis for the incidence of TB illustrated that unexposed patients had more chance of developing TB than exposed patients (*p* < 0.0001) (Figure 2).

**Table 3:** One-way ANOVA result of CD4 cells among patients living with HIV/AIDS taking IPT and ART versus ART alone in Northern Ethiopia, 2009–2017.

Exposure to IPT		Mean CD4 cells per cubic millimeter + SEM
Baseline	Unexposed group (on ART alone)	177.75 ± 3.24
	Exposed group (on IPT + ART)	194.14 ± 6.33^*∗*^
2009	Unexposed group (on ART alone)	186.12 ± 3.76
	Exposed group (on IPT + ART)	215.18 ± 6.22^*∗∗*^
2010	Unexposed group (on ART alone)	261.38 ± 4.77
	Exposed group (on IPT + ART)	306.7 ± 7.70^*∗*^
2011	Unexposed group (on ART alone)	354.33 ± 6.76
	Exposed group (on IPT + ART)	385.69 ± 10.61
2012	Unexposed group (on ART alone)	392.67 ± 6.74
	Exposed group (on IPT + ART)	412.66 ± 10.59
2013	Unexposed group (on ART alone)	442.15 ± 9.02
	Exposed group (on IPT + ART)	457.16 ± 11.51
2014	Unexposed group (on ART alone)	473.91 ± 9.78
	Exposed group (on IPT + ART)	498.38 ± 11.69
2015	Unexposed group (on ART alone)	479.97 ± 8.40
	Exposed group (on IPT + ART)	528.92 ± 12.54^*∗∗*^
2016	Unexposed group (on ART alone)	522.13 ± 9.34
	Exposed group (on IPT + ART)	536.84 ± 10.91
2017	Exposed group (on IPT + ART)	511.33 ± 9.00
	Unexposed group (on ART alone)	538.94 ± 11.05

^*∗*^Statistically significant *p* value < 0.05; ^*∗∗*^statistically significant *p* value < 0.01. Effect of IPT on all-cause mortality and associated factors.

**Table 4:** Results of Cox regression analysis for factors associated with survival status among patients living with HIV/AIDS taking IPT and ART versus ART alone in Northern Ethiopia, 2009–2017.

Variable		Survival status	Death, *n* (%)	CHR [95% CI]	*p* value	AHR [95% CI]	*p* value
		Alive, *n* (%)					
Sex	Female	1,076 (94.4)	64 (5.6)	0.465 [0.336–0.643]	<0.0001^*∗∗*^	0.691 [0.495–0.964]	0.030^*∗*^
	Male	638 (88.2)	85 (11.8)	Reference		Reference	Reference
Residence	Urban	1,161 (93.0)	87 (7.0)	0.680 [0.491–0.941]	0.020^*∗*^	0.595 [0.427–0.831]	0.002
	Rural	553 (89.9)	62 (10.1)	Reference		Reference	
Scheduling	Unscheduled	641 (89.0)	79 (11.0)	0.712 [0.606–0.837]	0.0001^*∗∗*^	1.601 [1.154–2.222]	0.005
	Scheduled	1,073 (93.9)	70 (6.1)	Reference		Reference	
Baseline functional status	Bedridden (2)	121 (88.3)	16 (11.7)	2.163 [1.255–3.726]	0.005	1.617 [0.935–2.796]	0.086
	Ambulatory (1)	415 (86.6)	64 (13.4)	2.515 [1.790–3.534]	<0.0001^*∗∗*^	2.324 [1.643–3.288]	<0.0001^*∗∗*^
	Working	1,178 (94.5)	69 (5.5)	Reference		Reference	
CD4+ T cells (in cells/mm^3^)	≥250	1,318 (96.1)	54 (3.9)	0.176 [0.126–0.246]	<0.0001^*∗∗*^	0.217 [0.154–0.307]	<0.0001^*∗∗*^
	<250	396 (80.7)	95 (19.3)	Reference	<0.0001^*∗∗*^	Reference	
Exposure to IPT	No	1113 (89.6)	129 (10.4)	Reference		Reference	
	Yes	601 (96.8)	20 (3.2)	0.296 [0.185–0.474]	<0.0001^*∗∗*^	0.339 [0.211–0.545]	<0.0001^*∗∗*^

^*∗*^Statistically significant at*p* < 0.05; ^*∗∗∗*^statistically significant at *p* < 0.0001. The following factors were analyzed at the crude stage: sex, age, place of residence, scheduling, baseline functional status, specific ARV regimen, body mass index, exposure variable, and WHO clinical staging. AHR: adjusted hazard ratio; ARV: antiretroviral regimen; BMI: body mass index; CD4^+^ T cells: cluster of differentiation 4 positive T cells; CI: confidence interval; CHR: crude hazard ratio; WHO: World Health Organization.

## Data Availability

The datasets supporting the conclusions of the study are included within the manuscript. Any additional data will be available on request by contacting the corresponding author through tesfay.mehari@mu.edu.et.

## Additional Points

*Strengths and Limitations of This Study.* (i) This study was adequately powered to study the relationship between the effect of ART used alone and with IPT. (ii) This study employed a robust sample size and adequate number of events. (iii) TB diagnosis was presumptive or was based on sputum and chest X-ray results in most cases, which might have led to over- or underestimation of the actual number of TB cases. (iv) We excluded patients who were lost to follow-up, and these patients may have died and had lower CD4 counts and more advanced World Health Organization (WHO) disease stage putting them at a greater risk for TB as compared to those active in follow-up. (v) The study did not include variables such as anemia and other comorbidities in our analyses because of missing data.

## Supplementary Materials

Supplementary Materials Supplementary Figure 1. Numbers of target and study population and inclusion and exclusion criteria of the study patients in Tigray Region. Supplementary Figure 2. Log minus log function for assumption of the proportional hazard. Supplementary Figure 3. Trend of weight (A) and body mass index (B) among patients living with HIV/AIDS taking IPT and ART versus ART alone in Northern Ethiopia, 2009–2017. Supplementary Figure 4. Kaplan–Meier survival analysis for patients living with HIV/AIDS taking IPT and ART versus ART alone in Northern Ethiopia, 2009–2017.
