# Supplementary material for: Does Isoniazid Preventive Therapy Provide Better Treatment Outcomes in HIV-Infected Individuals in Northern Ethiopia? A Retrospective Cohort Study
Source: AIDS Res Treat. 2020 Jan 21;2020:7025738. doi: 10.1155/2020/7025738 (PMC7204289; doi:10.1155/2020/7025738)
Supplement: Supplementary Materials — Supplementary Figure 1. Numbers of target and study population and inclusion and exclusion criteria of the study patients in Tigray Region. Supplementary Figure 2. Log minus log function for assumption of the proportional hazard. Supplementary Figure 3. Trend of weight (A) and body mass index (B) among patients living with HIV/AIDS taking IPT and ART versus ART alone in Northern Ethiopia, 2009–2017. Supplementary Figure 4. Kaplan–Meier survival analysis for patients living with HIV/AIDS taking IPT and ART versus ART alone in Northern Ethiopia, 2009–2017. [file 7025738.f1.docx]

**Supplementary Figure 1. Numbers of target and study population, and inclusion and exclusion criteria of the study patients in Tigray Region.**


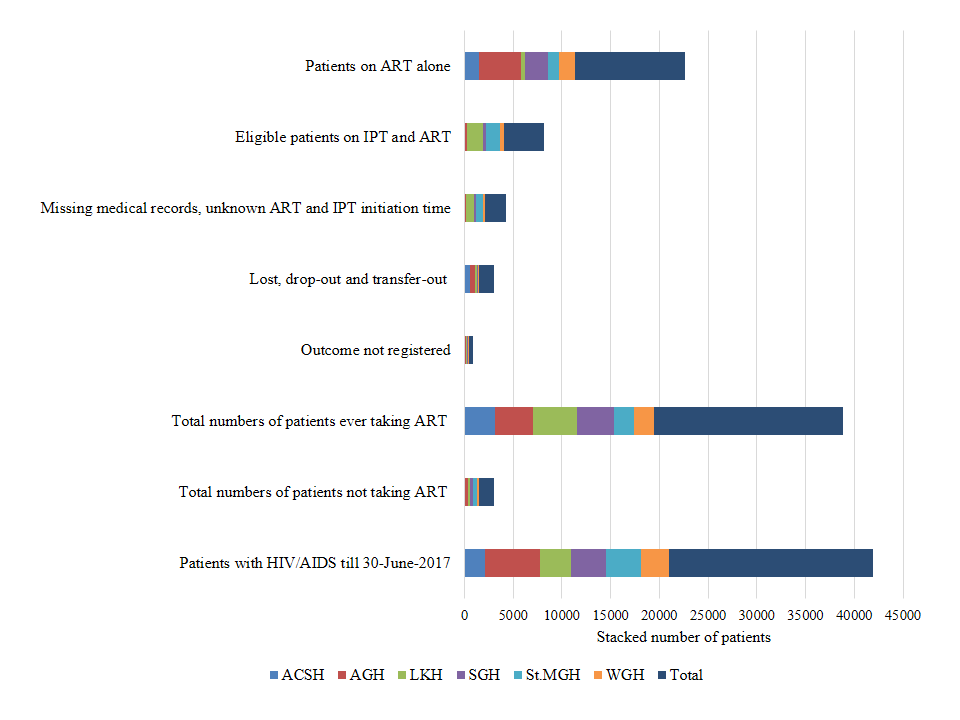


ACSH=Ayder Comprehensive Specialized Hospital; AGH=Alamata General Hospital; LKH=Lemlem Karl Hospital; SGH=Suhul General Hospital; St.MGH=Saint Marry General Hospital; WGH=Wukro General Hospital.

Supplementary Figure 1 indicates the total numbers of target and study population, and inclusion and exclusion criteria of the study patients in Tigray Region for the study period – further disaggregated by the six study settings.

**Supplementary Figure 2. Log minus log function for assumption of the proportional hazard**


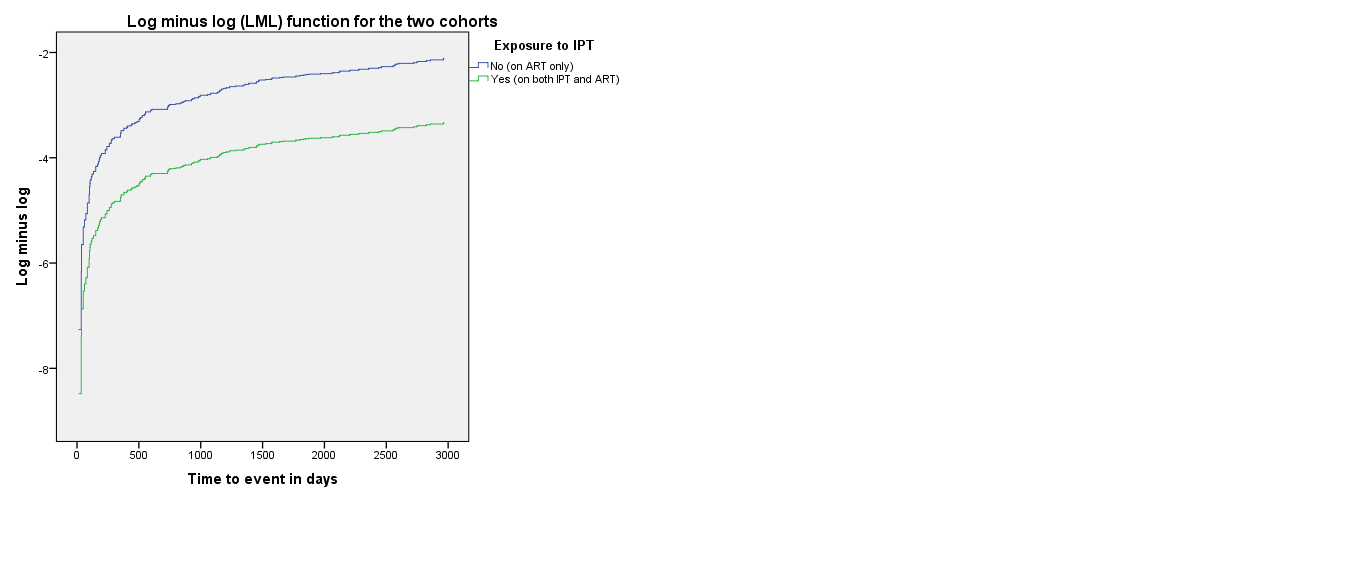


Supplementary Figure 2 depicts the graph of log minus log survival curve plotted against time to event (in days) which was used as a graphical representation for the assumption of proportional hazard.

**Supplementary Figure 3. Trend of weight (A) and body mass index (B) among patients living with HIV/AIDS taking IPT and ART versus ART alone in Northern Ethiopia, 2009–2017.**

| **A**   |
| --- |
| **B**   |

**Abbreviations**: AIDS: acquired immune deficiency syndrome; ART: antiretroviral therapy; BMI: body mass index; HIV: human immunodeficiency virus; IPT: isoniazid preventive therapy.

Supplementary Figure 3 shows the trend of weight (A) and body mass index (B) among patients living with HIV/AIDS taking IPT and ART versus ART alone in Northern Ethiopia, 2009–2017. The average baseline body weight was almost similar in both groups: exposed patients (49.8 kg) and unexposed patients (49.2 kg). The average baseline BMI was also about 19.8 kg/m2 for both cohorts. Over the nine-year period, the weight was increased by about 6.6 kg for both cohorts; and the BMI was increased by 2.8 kg/m2 for the exposed group and by 3.8 kg/m2 for the unexposed group, interpreting into about 15% and 16% increase from baseline to 2017, respectively.

**Supplementary Figure 4. Kaplan-Meier survival analysis for patients living with HIV/AIDS taking IPT and ART versus ART alone in Northern Ethiopia, 2009–2017.**

| **4A. Wukro General Hospital**  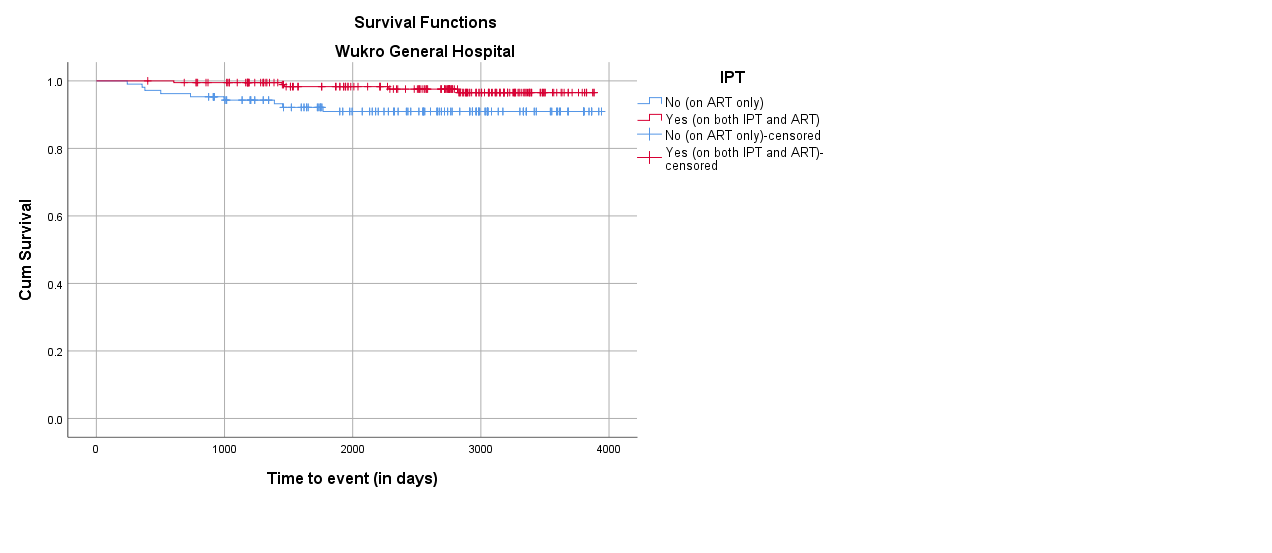 | **4B. Alamata General Hospital**  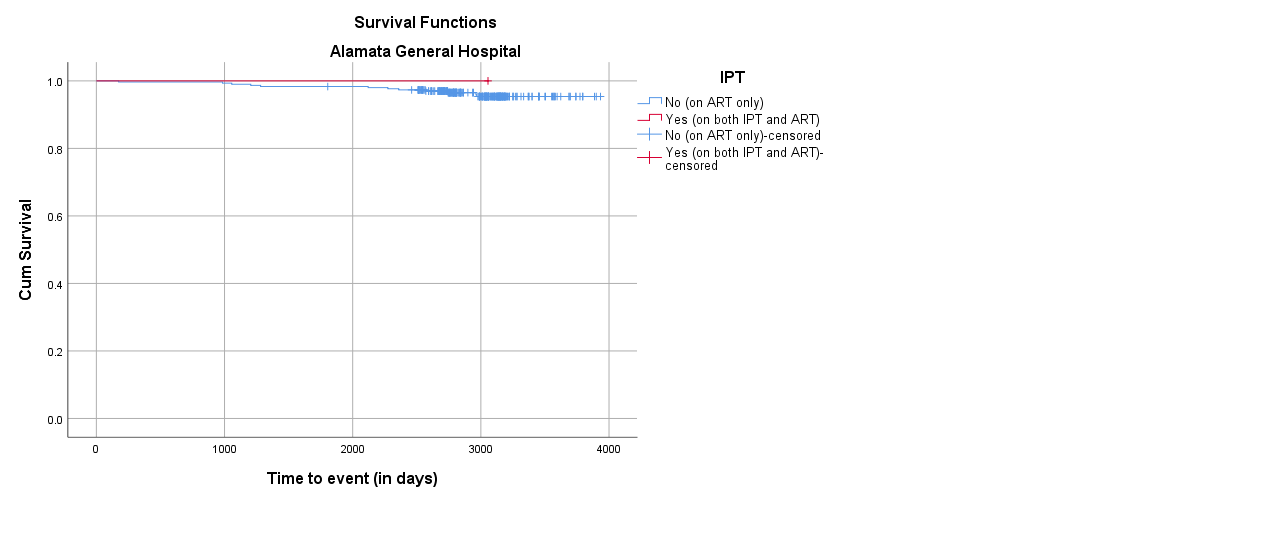 |
| --- | --- |
| **4C. St. Marry General Hospital**  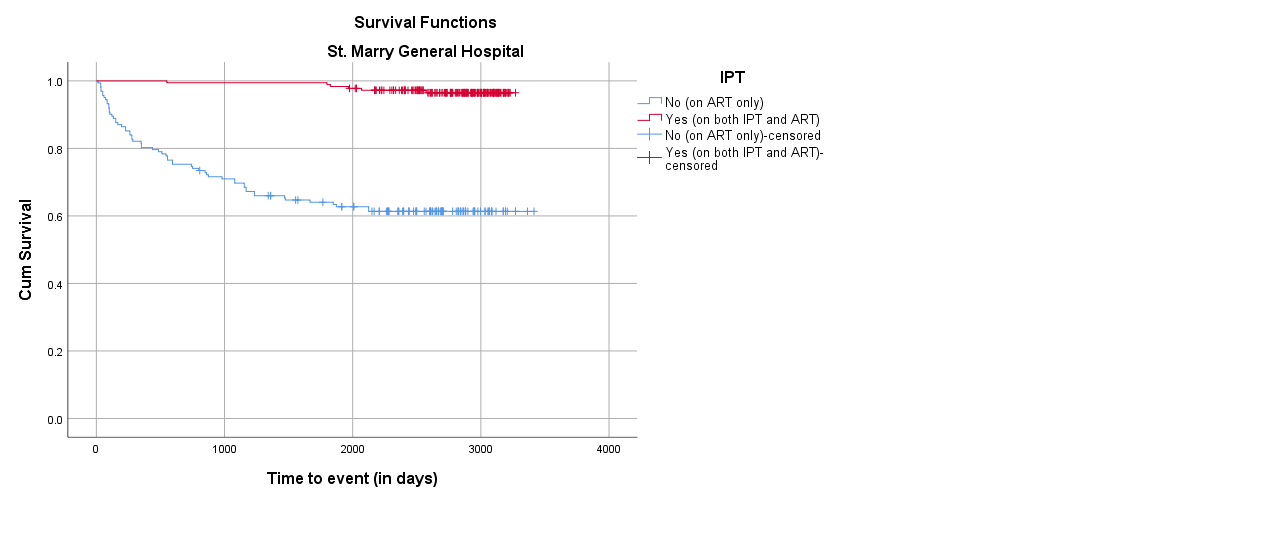 | **4D. Lemlem Karl General Hospital**  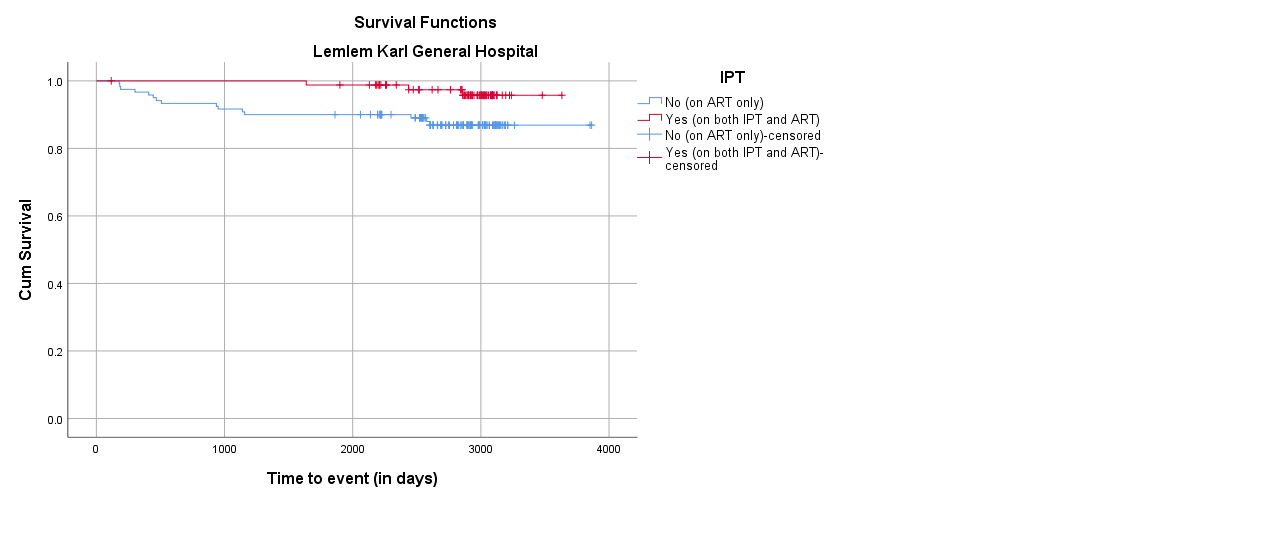 |
| **4E. Ayder Comprehensive Specialized Hospital**  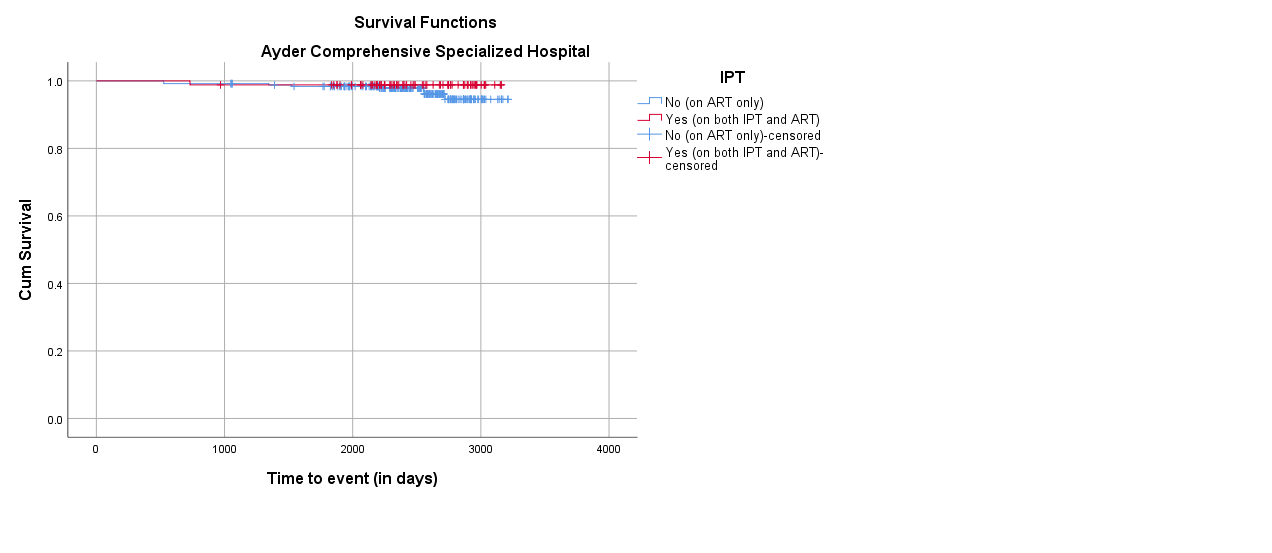 | **4F. Kahsay Abera General Hospital**  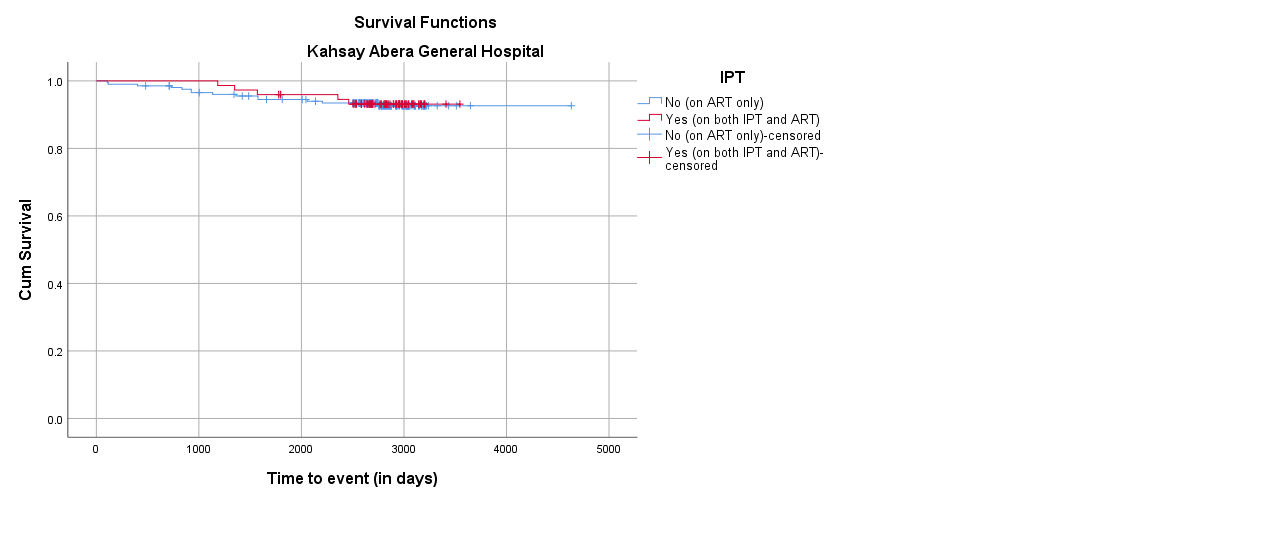 |

Supplementary Figure 4 shows the Kaplan-Meier (KM) survival analysis for patients living with HIV/AIDS taking IPT and ART versus ART alone in Northern Ethiopia, 2009–2017. The survival analysis is further stratified by the six study hospital settings (4A–4F). The HIV/AIDS treatment service provided in the six ART clinics is assumed to be similar among the hospitals. Any discrepancy shown in the KM graph might be related to the random selection of the exposed and unexposed groups from the general population. The overall Log Rank (Mantel-Cox) results for the six study settings showed that exposed groups had a better survival chance than unexposed groups at all levels of time (χ^2^=67.63, *p*<0.001).

# Appendix

**Propensity score matching**

Using a logistic regression analysis, the expected probability of starting IPT was calculated for each member of the cohort using a full list of covariates and interactions; this probability was considered the propensity score for starting IPT. Using this score, patients who were on IPT were matched with those patients who were not on IPT in a one-to-one manner according to the propensity scores. After matching the number of patients, the clinical characteristics between the matched pairs were then compared to ensure balance. In this propensity-matched cohort, the KM analyses were repeated to estimate covariate adjusted survival status between the groups.

The following variables showed a statistically significant correlation with the follow-up CD4^+^ T cells per cubic millimeter during 2017, after analyzing them through bivariate correlation analysis. Accordingly, the best predictors for the outcome variables were age; average scheduling status of the patient; baseline schedule status; scheduling status during 2014, 2015 and 2017; months on ART spanning from 2010−2017; average weight and the recorded weight across the period; baseline and average BMI, and BMI spanning from 2009−2013, 2016 and 2017 and number of CD4^+^ T cells per cubic millimeter across the period.

All these variables were then analyzed through binary logistic regression analysis and the predicted probability values (with 49 number of effects in the model) were then obtained. Following this, the mean predicted value were then calculated using Generalized Linear Models – with a binomial probability distribution and Logit Link Function – using the above predictors. The predicted values were the computed given a generation of random numbers. Lastly, an independent samples t-test was employed to compare the follow-up CD4^+^ T cells per cubic millimeter during 2017 between the two cohorts.

# Reporting checklist for cohort study.

|  |  | Reporting Item | Page Number |
| --- | --- | --- | --- |
| Title | [#1a](https://www.goodreports.org/strobe-cohort/info/#1a) | Indicate the study’s design with a commonly used term in the title or the abstract | 1 and 2 |
| Abstract | [#1b](https://www.goodreports.org/strobe-cohort/info/#1b) | Provide in the abstract an informative and balanced summary of what was done and what was found | 2 |
| Background / rationale | [#2](https://www.goodreports.org/strobe-cohort/info/#2) | Explain the scientific background and rationale for the investigation being reported | 4 and 5 |
| Objectives | [#3](https://www.goodreports.org/strobe-cohort/info/#3) | State specific objectives, including any prespecified hypotheses | 5 |
| Study design | [#4](https://www.goodreports.org/strobe-cohort/info/#4) | Present key elements of study design early in the paper | 5 and 6 |
| Setting | [#5](https://www.goodreports.org/strobe-cohort/info/#5) | Describe the setting, locations, and relevant dates, including periods of recruitment, exposure, follow-up, and data collection | 5 and 6 |
| Eligibility criteria | [#6a](https://www.goodreports.org/strobe-cohort/info/#6a) | Give the eligibility criteria, and the sources and methods of selection of participants. Describe methods of follow-up. | 6 and 7 |
|  | [#6b](https://www.goodreports.org/strobe-cohort/info/#6b) | For matched studies, give matching criteria and number of exposed and unexposed | 6 |
| Variables | [#7](https://www.goodreports.org/strobe-cohort/info/#7) | Clearly define all outcomes, exposures, predictors, potential confounders, and effect modifiers. Give diagnostic criteria, if applicable | 7 |
| Data sources / measurement | [#8](https://www.goodreports.org/strobe-cohort/info/#8) | For each variable of interest give sources of data and details of methods of assessment (measurement). Describe comparability of assessment methods if there is more than one group. Give information separately for for exposed and unexposed groups if applicable. | 6 and 7 |
| Bias | [#9](https://www.goodreports.org/strobe-cohort/info/#9) | Describe any efforts to address potential sources of bias | 7 |
| Study size | [#10](https://www.goodreports.org/strobe-cohort/info/#10) | Explain how the study size was arrived at | 6 |
| Quantitative variables | [#11](https://www.goodreports.org/strobe-cohort/info/#11) | Explain how quantitative variables were handled in the analyses. If applicable, describe which groupings were chosen, and why | 7 |
| Statistical methods | [#12a](https://www.goodreports.org/strobe-cohort/info/#12a) | Describe all statistical methods, including those used to control for confounding | 7 |
|  | [#12b](https://www.goodreports.org/strobe-cohort/info/#12b) | Describe any methods used to examine subgroups and interactions | 7 |
|  | [#12c](https://www.goodreports.org/strobe-cohort/info/#12c) | Explain how missing data were addressed | 7 |
|  | [#12d](https://www.goodreports.org/strobe-cohort/info/#12d) | If applicable, explain how loss to follow-up was addressed | NA |
|  | [#12e](https://www.goodreports.org/strobe-cohort/info/#12e) | Describe any sensitivity analyses | 7 |
| Participants | [#13a](https://www.goodreports.org/strobe-cohort/info/#13a) | Report numbers of individuals at each stage of study—eg numbers potentially eligible, examined for eligibility, confirmed eligible, included in the study, completing follow-up, and analysed. Give information separately for for exposed and unexposed groups if applicable. | Figure 1 |
|  | [#13b](https://www.goodreports.org/strobe-cohort/info/#13b) | Give reasons for non-participation at each stage | Figure 1 |
|  | [#13c](https://www.goodreports.org/strobe-cohort/info/#13c) | Consider use of a flow diagram | Figure 1 |
| Descriptive data | [#14a](https://www.goodreports.org/strobe-cohort/info/#14a) | Give characteristics of study participants (eg demographic, clinical, social) and information on exposures and potential confounders. Give information separately for exposed and unexposed groups if applicable. | 8 and Figure 2 |
|  | [#14b](https://www.goodreports.org/strobe-cohort/info/#14b) | Indicate number of participants with missing data for each variable of interest | Figure 1 |
|  | [#14c](https://www.goodreports.org/strobe-cohort/info/#14c) | Summarise follow-up time (eg, average and total amount) | 8 and 9 |
| Outcome data | [#15](https://www.goodreports.org/strobe-cohort/info/#15) | Report numbers of outcome events or summary measures over time. Give information separately for exposed and unexposed groups if applicable. | 8 to 10 |
| Main results | [#16a](https://www.goodreports.org/strobe-cohort/info/#16a) | Give unadjusted estimates and, if applicable, confounder-adjusted estimates and their precision (eg, 95% confidence interval). Make clear which confounders were adjusted for and why they were included | 11 |
|  | [#16b](https://www.goodreports.org/strobe-cohort/info/#16b) | Report category boundaries when continuous variables were categorized | 11 |
|  | [#16c](https://www.goodreports.org/strobe-cohort/info/#16c) | If relevant, consider translating estimates of relative risk into absolute risk for a meaningful time period | NA |
| Other analyses | [#17](https://www.goodreports.org/strobe-cohort/info/#17) | Report other analyses done—e.g., analyses of subgroups and interactions, and sensitivity analyses | 11 and 12 |
| Key results | [#18](https://www.goodreports.org/strobe-cohort/info/#18) | Summarise key results with reference to study objectives | 11, 12 |
| Limitations | [#19](https://www.goodreports.org/strobe-cohort/info/#19) | Discuss limitations of the study, taking into account sources of potential bias or imprecision. Discuss both direction and magnitude of any potential bias. | 3 |
| Interpretation | [#20](https://www.goodreports.org/strobe-cohort/info/#20) | Give a cautious overall interpretation considering objectives, limitations, multiplicity of analyses, results from similar studies, and other relevant evidence. | 12 to 14 |
| Generalisability | [#21](https://www.goodreports.org/strobe-cohort/info/#21) | Discuss the generalisability (external validity) of the study results | 12 to 14 |
| Funding | [#22](https://www.goodreports.org/strobe-cohort/info/#22) | Give the source of funding and the role of the funders for the present study and, if applicable, for the original study on which the present article is based | 8 |

The STROBE checklist is distributed under the terms of the Creative Commons Attribution License CC-BY. This checklist can be completed online using <https://www.goodreports.org/>, a tool made by the [EQUATOR Network](https://www.equator-network.org) in collaboration with [Penelope.ai](https://www.penelope.ai)
